# Leveraging machine learning in precision medicine to unveil organochlorine pesticides as predictive biomarkers for thyroid dysfunction

**DOI:** 10.1038/s41598-025-94827-z

**Published:** 2025-04-11

**Authors:** Samir Shamma, Mohamed Ali Hussein, Eslam M. A. El-Nahrery, Ahmed Shahat, Tamer Shoeib, Anwar Abdelnaser

**Affiliations:** 1 Institute of Global Health and Human Ecology, School of Sciences and Engineering, The American University in Cairo, New Cairo, 11835 Egypt; 2 Department of Chemistry, Faculty of Science, Suez University, Suez, Egypt; 3 Department of Chemistry, School of Sciences and Engineering, The American University in Cairo, Cairo, Egypt

**Keywords:** Organochlorine pesticides, Thyroid dysfunction, ML, RF, XGBoost, GBM, Biomarkers, Endocrinology, Thyroid hormones, Machine learning

## Abstract

Exposure to organochlorine pesticides (OCPs) poses significant health risks, including cancer, endocrine dysregulation, neurological disorders, and reproductive disruption. This study investigates the association between OCP exposure and thyroid disturbances using machine learning (ML) models. Blood samples were analyzed for the concentration of 16 OCPs and thyroid hormones (T3, T4, TSH) using traditional methods such as Logistic Regression and least absolute shrinkage and selection operator **(**LASSO) and more advanced ML models such as Random Forest (RF), Support Vector Machine (SVM), XGBoost, and Gradient Boosting Machine (GBM). High frequencies of OCPs, including Heptachlor, Heptachlor epoxide, γ-HCH, Aldrin, Endrin aldehyde, α-endosulfan, and Methoxychlor, were detected in over 70% of serum samples. The RF and GBM models achieved the highest accuracy at 90.91%, while XGBoost demonstrated a high ROC-AUC score of 94.02%. The SVM model also showed robust performance, whereas Logistic Regression exhibited weaker results. Our findings highlighted specific OCPs, such as Methoxychlor, p,p-DDT, Heptachlor, Endrin, and various HCH isomers, could impact thyroid function. The study supports a strong correlation between OCP exposure and thyroid dysfunction, demonstrating high accuracy in classifying thyroid status using ML models. Significant OCPs identified include *p*,* p*-DDT, Methoxychlor, Endrin, β-endosulfan, and Heptachlor, which are associated with thyroid dysfunction.

**Supplementary Information:**

The online version contains supplementary material available at 10.1038/s41598-025-94827-z.

## Introduction

Organochlorine pesticides (OCPs) are chlorine-atom-containing organic compounds broadly used as anthropogenic pesticides in the mid-20th century^1^. They are famous for their endurance within the ecosystem, mostly stemming from their chemical stability, extended half-life, and resistance to degradation. These properties facilitate their accumulation in soils, water systems, ecosystems, and food chains for an extended period, reaching decades and imposing a high risk of adverse health outcomes^2–4^. Along with their environmental persistence, OCPs’ lipophilicity renders them stored in fatty tissues, allowing for bioaccumulation and biomagnification. Consequently, causing ecosystem disruption and increasing the risk of diseases such as cancer, endocrine disruption, neurological disorder, and reproductive issues^4–9^. OCPs are constrained by regulatory bodies such as the Stockholm Convention, The Environmental Protection Agency (EPA), and the World Health Organization (WHO)^2,4,10,11^.

One significant health risk of OCPs exposure is endocrine dysregulation. Among endocrine pathways, thyroid dysfunction has been increasingly associated with OCP exposure, yet inconsistencies remain in the literature^12^. A plethora of evidence substantiates the correlation between OCPs exposure and variation in thyroid hormone levels, specifically triiodothyronine (T3), thyroxine (T4), and thyroid-stimulating hormone (TSH) in adults and children^13,14^. For instance, exposure to *p*,* p*-DDE in rats results in significantly reduced serum total T4 and free T4, accompanied by a decline in transthyretin levels in serum and increased expression of thyroid hormone receptors (TRα1 and TRβ1) mRNA in the hypothalamus^14^. In addition, in vitro studies demonstrate that DDT inhibits the TSH receptor, decreasing T3 and T4 13 bioactivity. Besides, one study reported that HCB interference with the HPT axis was associated with reduced T3 and T4 activity and T4-to-T3 conversion^15^. Although numerous studies have shown that OCP exposure affects thyroid function, the degree and mechanisms of disruption remain debated, particularly in human populations^16–21^. Additionally, studies focused on the implications of prenatal exposure to OCPs have emphasized the link between OCPs exposure and altered thyroid hormone levels in newborns. They showed that exposure to varying concentrations of OCPs could alter thyroid hormone levels^22–25^. Yet, conflicting evidence persists, as some studies report no or weak associations with thyroid dysfunction. A meta-analysis indicates a significant association between OCP exposure and hypothyroidism. Still, it also highlights a lack of clear evidence linking general environmental exposure to thyroid dysfunction, often due to limitations in exposure assessment methods. This underscores the need for more robust, population-specific research that evaluates the direct impact of OCPs on thyroid function using objective biomarkers^26^.

Machine learning (ML) models have emerged in medicine, offering irreplaceable tools for disease diagnosis and classifications, which could enhance better and more accurate early disease diagnosis^27^. Recent studies have revealed that various ML and deep learning models can accurately predict thyroid dysfunctions and have been widely used for thyroid disease prediction and classifications^28,29^. Garcia et al. utilized various ML algorithms to predict the compounds that are most likely to affect thyroid dysfunctions. They found that thyroid peroxidase and thyroid hormone receptor (TR) had the highest predictive accuracy, with F1 scores of 0.83 and 0.81, respectively^30^.

Despite the recognized thyroid toxicity of OCPs, no study has comprehensively assessed the association between OCP exposure and thyroid homeostasis using ML models in an Egyptian cohort. Therefore, this work aims to evaluate the associations relating OCPs exposure to thyroid dysfunctions in the Egyptian cohort and employs several ML models to predict whether various OCPs contribute to thyroid homeostasis. Given the widespread historical use of OCPs in Egypt and their persistence in the environment, understanding their potential endocrine effects is critical for public health and regulatory policies. This is the first study to examine the association of 16 OCPs in the serum in the Egyptian cohort with thyroid dysfunctions and employ different ML algorithms for detecting probable OCPs associated with thyroid homeostasis.

## Results

### The concentration of OCPs in serum samples

Our study included 169 females and 140 males aged 12 to 84, with a mean age of 39.9. We examined the concentrations of 16 OCPs in our cohort’s serum. The 16 chosen OCPs can be categorized into three main groups: cyclodienes, HCHs, and DDTs. The median concentration were calculated for OCPs. For HCH isomers, the OCPs concentration in this group ranged from 0 to 4806 ng/g lipid, and β-HCH elicited the highest median concentration at 482 ng/g, followed by γ-HCH at 391 ng/g and α-HCH at 324 ng/g. In addition, the cyclodienes groups OCPs concentrations ranged from 0 to 5288 ng/g lipid. Endrin exhibited the highest median concentration at 315 ng/g, followed by Aldrin, α-Endosulfan, Endrin-aldehyde, β-Endosulfan, and Dieldrin at a median concentration of 313, 288, 282, 224, and193 ng/g lipid, respectively. Besides, for the DDTs group, the concentrations ranged from 0 to 3016 ng/g lipid with the methoxychlor followed by *p*,* p-*DDT showed the peak median concentration at 366 ng/g and 160 ng/g, respectively. Additionally, Table S6 summarizes each OCP’s minimum, maximum, mean, and median values. To further enhance our understanding of the potential effect of these OCPs on THs, we tested the thyroid hormone levels in 230 out of the 309 individuals included in our study. Our results demonstrated that 132 individuals had thyroid disturbances (61 had hypothyroidism, 71 had hyperthyroidism), and 98 had normal thyroid levels. Numerous OCPs were identified with high frequencies (Table S6). Among those OCPs, Heptachlor, Heptachlor epoxide, Aldrin, Endrin-aldehyde, γ-HCH, α-endosulfan, and Methoxychlor were identified in more than 70% of serum samples (*n* = 309). Only Endrin-aldehyde was observed in more than 90% of serum samples.

### Association between OCPs and hormone concentrations in serum

We further examined the correlation between OCPs concentration and thyroid hormones using Spearman’s correlation. Our data revealed a significant correlation between certain OCPs, T3, T4, and TSH, as depicted in Fig. S1. Specifically, TSH levels negatively correlated solely with endrin (*r* = −0.15, *P* = 0.04). Moreover, total T4 levels displayed a negative correlation with γ-HCH (*r* = −0.27, *P* = 0.04), dieldrin (*r* = −0.25, *P* = 0.04), Endrin-aldehyde (*r* = −0.27, *P* = 0.04), and α-endosulfan (*r* = −0.25, *P* = 0.03).

### Bivariate logistic regression model

Our study used the Logistic Regression Model to investigate the link between multiple OCPs exposure and thyroid status. The model achieved a 50% accuracy score, 55.56% precision, 76.92% recall rate, and 64.52% F1 score. However, the ROC-AUC of 56.41% suggests that the model cannot differentiate between the two classes, performing only marginally better than random chance.

**Fig. 1:**
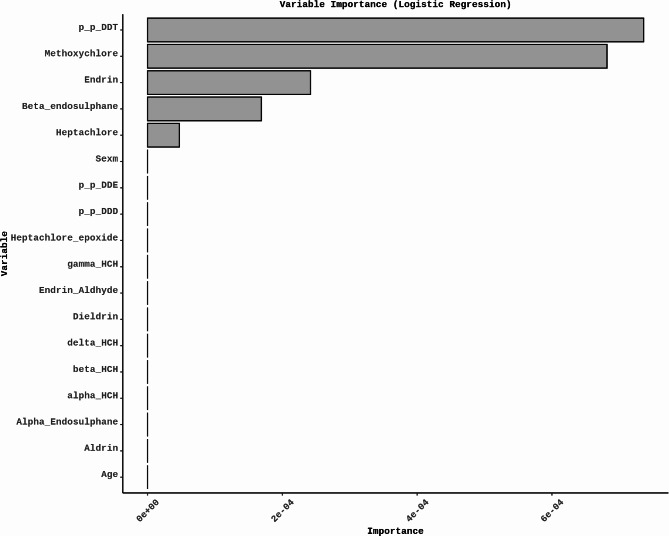
The most essential variable contributing to the classification of thyroid status in logistic regression with LASSO regularization is *p*,* p*-DDT, followed by Methoxychlor, Endrin, β-endosulfan, and Heptachlor.

The size of the coefficients indicates the variable’s importance. The most significant variables in classifying thyroid dysfunction were identified as *p*,* p*-DDT, Methoxychlor, Endrin, β-endosulfan, and Heptachlor (Fig. 1). Although the Logistic Regression model had a good recall and F1 Score, its precision and ROC-AUC indicate the potential for improvement. Hence, we incorporate more advanced modeling techniques to enhance the model’s ability to identify thyroid dysfunctions.

### Random forest model

**Fig. 2:**
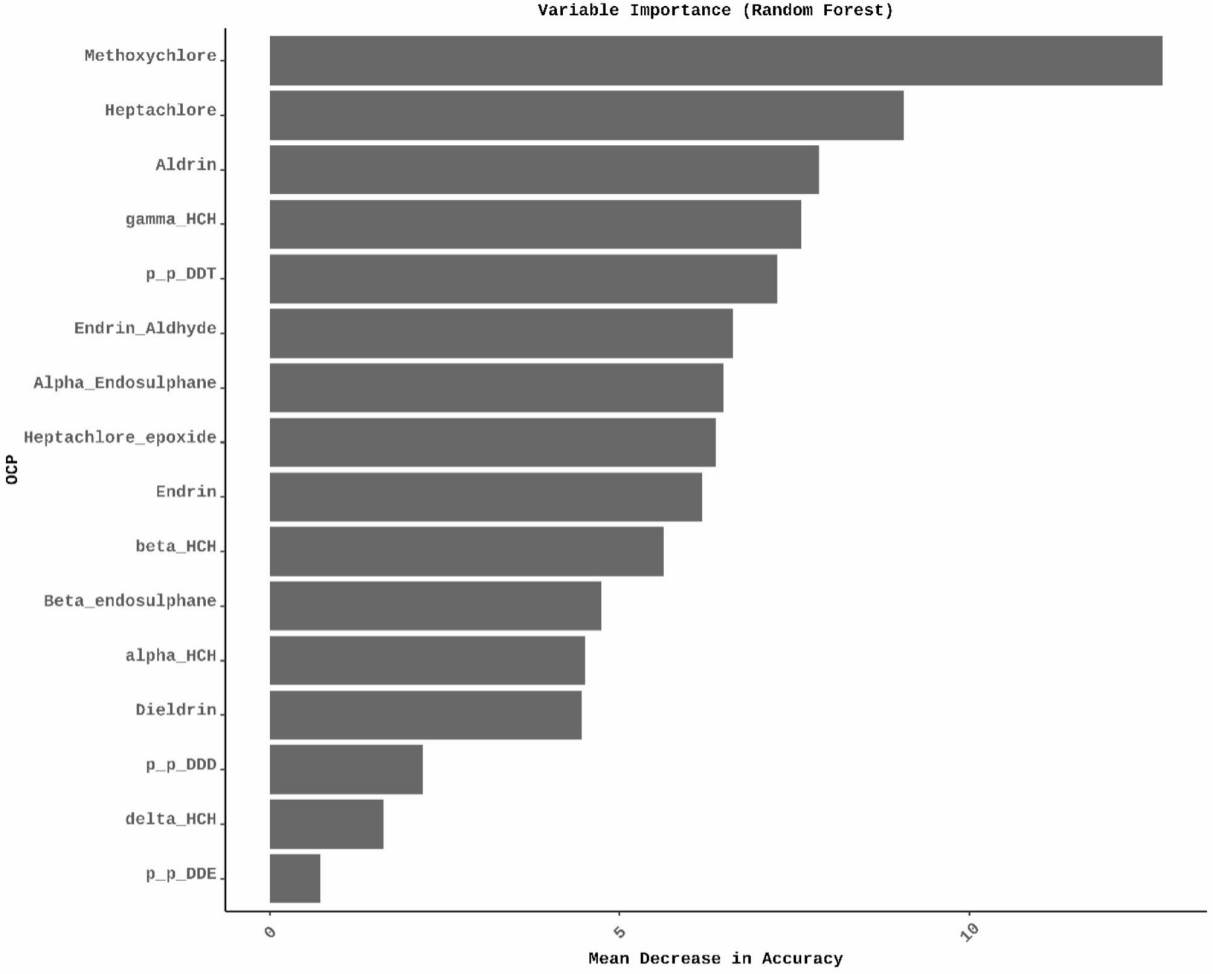
The most crucial variable contributing to the classification of thyroid status in RF is Methoxychlor followed by Heptachlor, Aldrin, γ-HCH, *p*,* p*-DDT, Endrin-aldehyde, α-endosulfan, Heptachlor-epoxide, Endrin, β-HCH, β-endosulfan, α-HCH, Dieldrin, *p*,* p*-DDD, δ-HCH, and *p*,* p*-DDE.

The results showed that as “mtry” increased, there was an improvement in ROC-AUC, reaching the highest value at “mtry” = 10, as shown in Table S3. The RF model performed well across various metrics. It achieved a 90.91% accuracy, 92.31% recall, and a 92.31% F1 Score, balancing precision and recall. Additionally, the ROC-AUC score of 89.74% demonstrates the model’s capability to effectively distinguish between ‘Normal’ and ‘Abnormal’ classes (Fig. 2).

Overall, the RF model reasonably classified thyroid status while maintaining a balanced trade-off between sensitivity and specificity. The variable importance analysis highlighted the critical predictors contributing to the model’s decisions, identifying the most significant variables in classifying thyroid dysfunction. These variables include Methoxychlor, Heptachlor, Aldrin, γ-HCH, *p*,* p*-DDT, Endrin-aldehyde, α-endosulfan, Heptachlor-epoxide, Endrin, β-HCH, β-endosulfan, α-HCH, Dieldrin, *p*,* p*-DDD, δ-HCH, and *p*,* p*-DDE, offering valuable information for clinical and research applications (Fig. 2).

### Support vector machine

The SVM model performed excellently with the best combination of sigma = 0.01 and C = 10. It showed 86.36% accuracy, 85.71% precision, a recall rate of 88.89%, and an F1-score of 88.89%. This indicates the model’s proficiency in effectively managing false positives and negatives. The model’s ROC-AUC of 89.74% indicates a high level of proficiency in differentiating between the two classes.

### Extreme gradient boosting

**Fig. 3:**
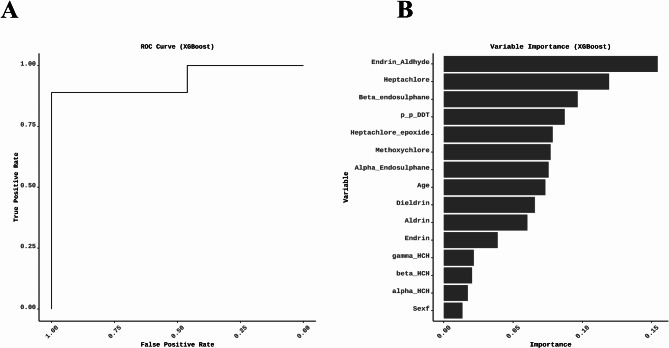
The XGBoost model ROC-AUC score and variable importance. (A) The ROC-AUC score is 94.02%, indicating the model’s strong capacity to effectively differentiate between Normal and Abnormal thyroid status. (B) The variable importance analysis of the XGBoost model shows that certain OCPs, including Endrin-aldehyde, Heptachlor, β-endosulfan, *p*,* p*-DDT, Heptachlor-epoxide, Methoxychlor, α-endosulfan, age, Dieldrin, Aldrin, Endrin, γ-HCH, β-HCH, and α-HCH, have a significant influence on the model’s decisions.

The XGBoost model showed 86.36% accuracy and 91.67% precision. The recall stood at 84.62%, and the F1 score was 88%, reflecting the model’s overall effectiveness. Notably, the ROC-AUC score was 94.02%, indicating the model’s strong ability to effectively differentiate between ‘Normal’ and ‘Abnormal’ classes (Fig. 3). These outcomes underscore the effectiveness of the XGBoost model in this context and highlight high precision and a robust ROC-AUC score, both critical for accurate classification. The variable importance analysis from the XGBoost model provides valuable insights into the predictors significantly influencing the model’s decisions, specifically Endrin-aldehyde, Heptachlor, β-endosulfan, *p*,* p*-DDT, Heptachlor-epoxide, Methoxychlor, α-endosulfan, age, Dieldrin, Aldrin, Endrin, γ-HCH, β-HCH, α-HCH (Fig. 3). This information is crucial for understanding the underlying patterns in the data. It can guide future research or interventions aimed at improving thyroid health.

### Gradient boosting machine

The GBM model demonstrated excellent predictive capabilities for determining thyroid status, achieving 90.91% accuracy. Furthermore, the model exhibited a remarkable precision rate of 92.31% with a recall rate of 92.31% and an F1-Score of 92.31%, highlighting the model’s high performance in classification tasks. Additionally, the GBM model achieved a ROC-AUC score of 88.03%, demonstrating its robust ability to differentiate between the ‘Normal’ and ‘Abnormal’ categories. These results highlight the GBM model’s robustness and dependability in predicting thyroid status, making it a valuable tool for medical research and potential clinical applications.

**Fig. 4:**
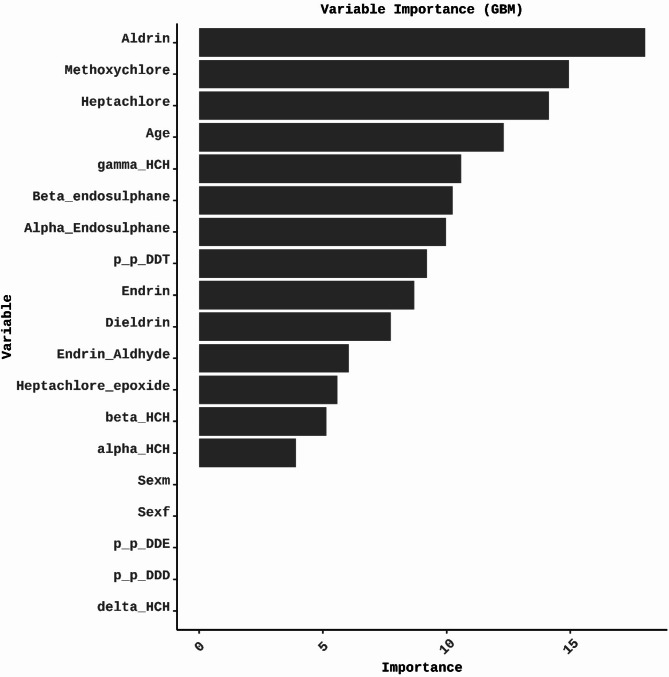
Illustrate the variable importance of the classification of thyroid status in the GBM model. The most crucial predictor is Methoxychlor Followed by *p*,* p*-DDT, Endrin-aldehyde, γ-HCH, age, α-endosulfan, β-HCH, Heptachlor-epoxide, Endrin, Dieldrin, Heptachlor, Aldrin, α-HCH, and β-endosulfan, in order of significance.

The variable importance analysis in GBM models identified the essential predictors that significantly contribute to the model’s decisions, particularly in categorizing thyroid dysfunction. The identified variables include Methoxychlor, *p*,* p*-DDT, Endrin-aldehyde, γ-HCH, age, α-endosulfan, β-HCH, Heptachlor-epoxide, Endrin, Dieldrin, Heptachlor, Aldrin, α-HCH, and β-endosulfan (Fig. 4).

## Discussion

The anthropogenic usage of OCPs in the mid-20th century for pest and disease control has significantly impacted the environment and posed serious risks to human health. Exposure to these chemicals has been linked to various diseases, including cancer, endocrine disruption, neurological disorders, and reproductive issues^4–9^. OCPs detection in our samples ranged from 5.5 to 92.4%, with the highest frequencies observed for Endrin-aldehyde, Heptachlor epoxide, and Methoxychlor, while p, p-DDE had the lowest detection rate (5.5%) (Table 1). The HCHs group showed a high detection rate (86.7%) and an average concentration of 2083 ng/g lipid, indicating widespread exposure. In contrast, p,p-DDE exhibited lower exposure levels with a detection rate of 5.5% and an average concentration of 14 ng/g lipid. Despite the long-standing ban on OCPs in Egypt, DDT remains detectable in water, vegetables, milk, fruits, fish, and sediments. A study on Manzala Lake reported high DDT concentrations, likely due to past agricultural use near the lake. Another study found that p, p-DDT, p,p-DDE, and dieldrin levels in the River Nile exceeded WHO guidelines^31^. Our findings align with previous studies showing significant variability in OCP concentrations across populations. Research suggests that cyclodienes are more prevalent than HCHs and DDTs in rural farming communities, whereas urban areas exhibit higher HCH levels than cyclodienes and DDTs^32,33^. These variations highlight the importance of considering geographical location, occupation, and lifestyle as cofactors when interpreting OCPs’ exposure levels.

**Table 1:** Different validation parameters for calibration curves, recovery rates, and detection rates of OCPs in serum samples (*n* = 309).

Compound	*R* ^2^	LOD (ppb)	LOQ (ppb)	Recovery % ± SD (50 ppb)	Detectable rate (%)	25% Percentile	75% Percentile
α-HCH	0.995	0.44	0.82	85.7 ± 4.43	61.4	0	1681
β-HCH	0.997	0.32	0.99	78.88 ± 3.73	68.9	0	1058
γ-HCH	0.995	0.41	0.90	84.22 ± 3.51	74.7	0	622.8
δ-HCH	0.999	0.32	0.99	82.42 ± 2.44	32.1	0	116
Heptachlor	0.995	0.44	0.91	78.26 ± 4.98	73.1	0	635.2
Heptachlor epoxide	0.995	0.26	0.63	71.74 ± 3.14	80.9	0	668.3
Aldrin	0.995	0.33	0.59	80.42 ± 1.77	77.6	136.2	615.8
Dieldrin	0.996	0.33	0.29	76.22 ± 1.56	67.8	0	460.3
Endrin	0.996	0.26	0.94	85.74 ± 5.40	69.2	0	663.9
Endrin-aldehyde	0.996	0.32	0.68	87.90 ± 4.22	92.4	35.68	564.7
α-endosulfan	0.995	0.30	0.98	74.88 ± 2.88	73.4	0	526.3
β-endosulfan	0.996	0.33	0.29	82.56 ± 2.98	60.5	0	473.8
*p*,* p* -DDD	0.999	0.39	0.82	85.98 ± 4.41	11.3	0	0
*p*,* p*-DDE	0.998	0.09	0.95	89.64 ± 3.95	5.5	0	0
*p*,* p*-DDT	0.995	0.09	0.19	77.84 ± 3.77	62.7	0	270.7
Methoxychlor	0.996	0.10	0.30	77.04 ± 5.04	80.5	144.2	750.1

We employed various ML algorithms to investigate the relationship between exposure to OCPs and thyroid dysregulation. Our findings provide valuable insights into the factors influencing thyroid disturbance and can help understand the intricate interaction between various OCPs and thyroid disturbances. Our analysis revealed that ensemble methods, mainly RF and GBM, consistently outperformed the other models, emphasizing their effectiveness in classification tasks. The XGBoost demonstrated an exceptional performance, especially in classifying cases. This was followed by the SVM model that showed a good balance of precision and recall, making it suitable for scenarios where both true favorable and true negative rates are essential. Conversely, Logistic Regression with LASSO regularization exhibited limited performance compared to other models, possibly due to assuming linearity and its inability to capture complex data patterns (Table 2).

**Table 2:** Comparative analysis of various ML models employed to classify the thyroid status.

Model	Accuracy	Precision	Recall	F1-Score	ROC-AUC
Logistic Regression	50.00%	55.56%	76.92%	64.52%	56.41%
RF	90.91%	92.31%	92.31%	92.31%	89.74%
SVM	86.36%	85.71%	92.31%	88.89%	89.74%
XGBoost	86.36%	91.67%	84.62%	88.00%	94.02%
GBM	90.91%	92.31%	92.31%	92.31%	88.03%

Furthermore, we examined the various variables that contribute to the classification of thyroid status and consequently contribute significantly to thyroid disturbances, as depicted in (Figs. 1, 2, 3 and 4**)**. Our results emphasized that there is a consistent presence of specific pesticides such as Methoxychlor, *p*,* p*-DDT, Heptachlor, Endrin, and various HCH isomers (γ-HCH, β-HCH, α-HCH) across different models, highlighting their potential impact on thyroid dysfunction. Our results align with previous research linking these pesticides to endocrine disruption, especially thyroid disturbances. For example, Methoxychlor has been shown to alter thyroid hormone metabolism by interfering with iodothyronine 5′-mono deiodinase, type I (5′-ID1), which is responsible for converting T4 to T3, implying that methoxychlor is a potential disruptor of THs metabolism^34^. Additionally, Freire et al. revealed that chronic exposure to methoxychlor is associated with the alteration of thyroid hormones in Brazilian populations^16,22^.

Moreover, our data suggested a weak association between OCPs exposure and thyroid hormone alteration, which aligned with the meta-analysis results indicating no association between environmental exposure and hypothyroidism^26^. In contrast, a study by Yamazaki et al. examined the connection relating OCPs exposure to thyroid hormone levels in 330 mothers and infant pairs, emphasizing a negative correlation between DDE, DDT, and dieldrin and maternal FT4 while a positive association between cis-nonachlor and mirex and infant FT4. This study supports the association between OCPs exposure and maternal and infant thyroid hormone levels. However, the small sample size and the fact that it included only a specific population significantly limited the results^35^. One study investigated the association between organohalogen contaminants, including OCPs, and thyroid hormone levels and reported a negative association between deiodinase and OCPs in East Greenland polar bears^36^. In addition, Nost et al. examined the concentrations of halogenated organic contaminants, including OCPs, in chicks of two seabird species and revealed lower concentrations than the previously reported results, as well as no correlation between OCPs and thyroid hormone concentrations^37^. These discrepancies in the reporting and contradictory results might be due to differences in the route and level of exposure between the same species as well as different species, intra-and interspecies variation in the main physiological, metabolic functions, stage of life, the genetic factors, multiple environmental co-exposures, and other study design used in various studies.

The inclusion of age as a feature in XGBoost and GBM models suggests that susceptibility to these substances may vary by age. Healthcare professionals should consider the potential influence of these pesticides on thyroid function, while regulatory authorities may need to reassess their safety. Further research is essential to understand how these pesticides affect thyroid function, requiring longitudinal and experimental investigations to establish causal connections and inform intervention strategies.

Nonetheless, our study has several limitations, including being cross-sectional, which inherently cannot determine causality. We have a small sample size of only 230 samples; therefore, we split our dataset into two 90% datasets for training and 10% of the dataset for testing the various ML models to overcome the limitation of sample size. Thus, extrapolation of our results to unforeseen data may be limited. We plan to increase the sample size to address this challenge in our future work. Additionally, further longitudinal studies with a large sample size should be conducted to determine a true causality to determine the causal relationship between OCPs and thyroid hormone disturbances. There might be various co-factors such as occupation, smoking, alcohol consumption, and diet. In addition, it was difficult to determine the mode of exposure to OCPs, whether from outdoor exposure, soil, food, or water. Moreover, this study focused primarily on age and sex as covariates due to their well-documented impact on thyroid function. However, other factors, such as lifestyle and health-related factors, might influence thyroid homeostasis. Further studies are warranted to examine the effects of these factors on OCPs-mediated thyroid dysfunction.

## Conclusion

Our research explores the connection between exposure to OCPs and adult thyroid hormone dysregulation. The study underscores the potential health risks posed by OCP exposure and its ability to disrupt thyroid function. Our ML algorithms revealed that the RF and GBM models achieved the highest accuracy at 90.91%, with equal precision, recall, and F1-Score at 92.31%. XGBoost also performed well, with a high ROC-AUC score of 94.02%. SVM demonstrated strong performance with an accuracy of 86.36% and a ROC-AUC of 89.74%.

On the other hand, Logistic Regression showed comparatively weaker performance, with 50% accuracy. Additionally, ensemble methods, mainly RF and GBM, outperformed other models. XGBoost also performed well, especially in classification tasks. SVM showed a good balance of precision and recall, while Logistic Regression exhibited limited performance. Our research identified significant pesticides such as *p*,* p*-DDT, Methoxychlor, Endrin, β-endosulfan, and Heptachlor as critical factors for categorizing thyroid dysfunction. Different models highlighted these pesticides’ importance and potential impact on thyroid disturbances, aligning with previous research on endocrine disruption.

## Materials and methods

### Sampling and preparation

In this cross-sectional study, we collected random blood samples from 309 adult Egyptian individuals undergoing blood analysis living in rural areas and suspected of being exposed to pesticides during hospital examinations. Between January and April 2022, blood samples were obtained from 169 females and 140 males aged 12 to 84 under fasting conditions. We examined the concentration levels of 16 OCPs in the blood. Out of 309 individuals, 79 were excluded from the analysis as their thyroid profile was incomplete. For the remaining 230 individuals, thyroid hormones were measured, including total or free T3, total or free T4, and TSH. We categorized samples into three groups: normal if they have no thyroid hormone disturbances or will be considered to have hypothyroidism if they have one of the following conditions: have high or low levels of THs according to one of the following criteria: patients who have low T3 (total T3 < 60 ng/dL or free T3 < 1.85 pg/mL) or low T4 (total T4 < 4.1 µg/dL or free T4 < 0.93 ng/dL) and high TSH (TSH > 4.2 mIU/L) or will be considered to have hyperthyroidism if they have one of the following conditions: high T3 (total T3 > 180 ng/dL or free T3 > 4.05 pg/mL) or high T4 (total T4 > 14 µg/dL or free T4 > 1.7 ng/dL) and low TSH (TSH < 0.5 mIU/L). The blood specimens were collected, centrifuged to separate the serum at 3000×g for 15 min, and kept at −20 °C for analysis.

### Chemicals and reagents

All target compounds were procured from SUPLECO (Bellefonte, PA) as certified standard OCPs mixture containing (α-HCH, β-HCH, γ-HCH, δ-HCH, aldrin, dieldrin, Endrin, Endrin-aldehyde, heptachlor, heptachlor epoxide, α-endosulfan, β-endosulfan, methoxychlor, *p*,* p*-DDE, *p*,* p*-DDD, *p*,* p*-DDT). Deuterated internal standards (HCH-d6, *p*,* p*- DDD –d6 and *p*,* p*- DDT –d6) were procured from Toronto Research Chemicals (North York, ON). HPLC grade solvents, such as acetonitrile (ACN), n-hexane, and methanol, utilized for sample analysis, were obtained from Fisher Scientific (Pittsburgh, PA). The Millipore Q Water Purification System was used for water purification (Millipore, Bedford, MA). QuEChERS tubes containing anhydrous magnesium sulfate (MgSO4) and sodium chloride (NaCl) were employed for salting out, and Primary secondary amine (PSA) that was utilized for sample cleanup was obtained from United Chemical Technologies (UCT, Bristol, Pennsylvania).

### Extraction and analytical analysis

To perform sample extraction and analytical analysis, we added 2 mL of ACN containing 50 µL of internal standards (IS) to a 15 mL centrifuge pre-packed tube with 800 mg MgSO4 and 200 mg NaCl. Subsequently, 1 mL of serum was added, then the sample was vigorously mixed to dissolve the IS, followed by continuous centrifugation at 4000 ×g for 5 min. The target compound in the upper layer was collected and transferred to a new 15 mL tube pre-packed with 180 mg PSA and 110 MgSO4. To clean up the sample, 2 mL of the extract was mixed and vortexed with PSA, followed by centrifugation at 4000×g for 4 min at 4ºC (Hermle Labortechnik GmbH, Wehingen, Germany). After that, the extract was transferred into the glass tube. Then, the sample was dried using a speed vac concentrator (Thermo Savant, Holbrook, NY). Finally, the resultant residue was reconstituted in 100 µL of n-hexane and injected into the GC–MS instrument for analysis.

Our analysis was performed using an Agilent 7890B gas chromatograph (Wilmington, DE) having Agilent 5977B mass spectrometric detection (Wilmington, DE) with an HP-5MS column (30 m ×0.25 mm internal diameter and 0.25 μm film thickness, J&W Scientific (Wilmington, DE). Analyses were carried out using helium (99.995%) as the carrier gas at a 1.0 mL/min flow rate at a splitless mode with an injection volume of 1 µL. The temperature program employed consisted of 160 °C for 4 min, followed by temperature ramping of 3 °C /min to 230 °C. The injector and detector were held at 250 °C and 260 °C, respectively. Mass spectra were obtained by electron ionization at 70 eV with a solvent delay of 6 min, having the source and quadrupole set at 230 °C and 150 °C, respectively. For quantification purposes, calibration curves consisting of 6 concentrations for each identified OCP were constructed using the OCPs/ standard mixture. The limit of detection (LOD) and quantification (LOQ) for each OCP compound ranged between 0.096 and 0.44 ng/mL and 0.199–0.996 ng/mL, respectively. Additionally, the recovery percentages of the solvent-spiked samples for 50 ng/mL OCPs ranged from 70.44 to 89.64, as depicted in Table 1.

### Total serum lipids concentration and lipid standardization

 The lipid weight of the serum normalized the concentrations of OCPs. Total cholesterol (mg/dL) and triglyceride (mg/dL) were analyzed by using the Advia 2400 instrument (Siemens Healthcare Diagnostics Inc, Deerfield, IL, US) to calculate the total serum lipids that required for lipid standardization of the OCPs measurements, and the following formula was utilized^38,39^:

$$Total\;lipid = \left[ {Total\;cholesterol\;({\text{mg}}/{\text{dl}}) \times 2.27} \right] + Triglycerides\;({\text{mg}}/{\text{dl}}) + 62.3\;{\text{mg}}/{\text{dL}}$$

### Hormonal profiling

To measure the serum concentrations of thyroid hormones, including free T3 (FT3), free T4 (FT4), total T3 (TT3), total T4 (TT4), and TSH, we used the ADVIA Centaur automatic Chemiluminescence Immunoassay Analyzer (Siemens Healthineers, Erlangen, Germany). Determinations and quality control were performed as per the manufacturer’s instructions manual. The assay ranges were 0.20–20.00 pg/mL for FT3, 20–651 ng/dL for TT3, 0.14–11.1 ng/dL for FT4, 0.42–24.86 µg/dL for TT4, and 0.005–160.03 µIU/mL for TSH, with sensitivity verified by detection limits (LOD) of 0.10 pg/mL for FT3, 10 ng/dL for TT3, 0.10 ng/dL for FT4, 0.30 µg/dL for TT4, and 0.005 µIU/mL for TSH, ensuring reliable detection of low concentrations. Accuracy was confirmed using quality control serum samples, with observed values within ± 10% of expected values. At the same time, precision assessments through intra-assay and inter-assay variability revealed coefficients of variation (CVs) below 6% for all analytes, demonstrating high reliability and repeatability in the measurements. In addition, our study assesses the concentrations of OCPs based on their lipid in ng/g fat, as these compounds are primarily stored in body fat. The recoveries of OCPs were found to be consistent and within acceptable limits; thus, adjustments for recovery were not made. In addition, Spearman’s correlation to examine the correlation between OCPs and thyroid hormones (Fig. S1).

### Data collection and preprocessing

Our dataset comprises 19 features, including thyroid status, age, sex, and 16 OCPs, including α-HCH, β-HCH, γ-HCH, δ-HCH, heptachlor, heptachlor-epoxide, Aldrin, Dieldrin, Endrin, Endrin-Aldhyde, α-Endosulphane, β-endosulfan, *p*,* p*-DDD, *p*,* p*-DDE, *p*,* p*-DDT, and Methoxychlor of 230 samples from the Egyptian adult cohort. The dependent variable has been classified into two classes: normal and abnormal. Our predictor variable is the concentrations of various OCPs in the blood of 230 patients, as depicted in Table S1. Values below the LOD were replaced with the $\:\:\text{LOD}/\:\surd\:2$^40^. Sex data was encoded as a factor with two levels: " f " for females and “m” for males. In addition, the thyroid status has two levels: normal and abnormal. Our data sample attributes and descriptions for each attribute and their data type are illustrated in Table S2. Due to our small sample size of only 230 samples, we split our dataset into two 90% datasets for training and 10% of the dataset for testing to overcome the limitation of sample size. Thus, extrapolation of our results to unforeseen data may be limited. We plan to increase the sample size to address this challenge in our future work. The predictors we looked at included thyroid status (dependent variable) and OCPs like α-HCH, β-HCH, γ-HCH, δ-HCH, Heptachlor, Heptachlor-epoxide, Aldrin, Dieldrin, Endrin, Endrin-Aldhyde, α-endosulfan, β-endosulfan, *p*,* p*-DDD, *p*,* p*-DDE, *p*,* p*-DDT, and Methoxychlor as independent variables. After conducting a more in-depth analysis of the model coefficients, we determined the critical predictors by considering their *P*-values. This allowed us to assess the importance of the features. We evaluated the model’s performance on the test by employing metrics like accuracy, precision, recall, F1-score, and ROC-AUC. Finally, we assessed the model’s robustness by comparing its performance during training and validation.

### Logistic regression with LASSO regularization

A Logistic Regression model is a supervised model that can be used when the dependent variable is binary to predict the probability of a binary outcome or observation occurring^41^. We employed the Logistic Regression model with the least absolute shrinkage and selection operator (LASSO) regularization through the “glmnet” package. To mitigate overfitting, the regularization parameter “lambda” was fine-tuned using 10-fold cross-validation using the “cv. “glmnet” function. We optimized the regularization parameter lambda through cross-validation to prevent overfitting (Table S5).

### Model selection and training

#### Random forest

The Random Forest (RF) model is an ensemble technique for classification and regression tasks. This model combines the output of various decision trees to produce a single result^42^. The stratified sampling technique was utilized in the RF model, employing the “randomForest” package. A 10-fold cross-validation was used to validate the model. Subsequently, the grid search explores the number of variables randomly selected as candidates at each split (mtry). The grid search examined values of (mtry) ranging from 2 to 12 (Table S3).

#### Support vector machine

The SVM model is a supervised ML method that performs classification and regression tasks. It can determine the optimal hyperplane in an N-dimensional space, separating data points into distinct classes within the feature space^43^. The SVM model uses a radial basis function (“RBF”) kernel using the “kernel” package. The hyperparameter tuning involved a grid search over the cost parameter (C) and the kernel width (“sigma”). The grid search explored values of “C” as [0.1, 1, 10] and “sigma” as [0.01, 0.05, 0.1] (Table S4). Then, we utilized 10-fold cross-validation for model validation and conducted a grid search to adjust hyperparameters, focusing on the kernel parameters (“RBF”) and the (“C”) (Table S5).

#### Extreme gradient boosting

XGBoost is a top-performance and effective ML model for regression and classification problems. It employs a gradient-boosting decision tree algorithm^44^. The XGBoost model was constructed using the " xgboost " package. We thoroughly evaluated our model’s performance by employing 10-fold cross-validation to determine the most suitable values for each hyperparameter. To ensure robustness, 10-fold cross-validation was used for performance assessment, resulting in balanced sample sizes of 187 or 188 for each fold. The hyperparameters of the XGBoost model were selected after a comprehensive grid search. The final parameters are as follows: 300 boosting rounds (nrounds), a maximum tree depth (max_depth) of 3, a learning rate (eta) of 0.3, a minimum loss reduction (gamma) of 5, a column subsample ratio (colsample_bytree) of 0.5, a minimum sum of instance weight in a child (min_child_weight) of 5, and a subsample ratio of 0.5 (Table S5).

#### Gradient boosting machine

The GBM is an ensemble model for regression and classification that uses gradient boosting, resulting in a more accurate model than other models^43^. The GBM model was implemented using the “gbm” package. We performed hyperparameter tuning over grid search using the following parameters: number of trees (“n.trees”), interaction depth (“interaction. depth”), learning rate (“shrinkage”), and the minimum number of observations in the terminal nodes (“n.minobsinnode”). Additionally, we thoroughly evaluated our model’s performance by employing 10-fold cross-validation to determine the most suitable values for each hyperparameter. Collectively, we used rigorous model development and validation procedures to mitigate bias and variance concerns. We provided extensive performance metrics to select the optimal model for predicting thyroid status based on OCP exposure, as summarized in Table S5.

#### Experimental setup and statistical analysis

The statistical analyses were performed using the GraphPad Prism 10.0 software for descriptive statistics. The statistical significance set to a *P*-value < 0.05 was chosen to represent statistically significant results. We evaluated the effectiveness of our ML models by quantifying their accuracy, precision, recall, F1-score, and ROC-AUC metrics. In addition, each model was assessed using a confusion matrix. We employed the Google Colab Notebook and executed the suggested methodology utilizing R language version 4.4.1 (2024-06-14).

## Electronic Supplementary Material

Below is the link to the electronic supplementary material.

Supplementary Material 1

## Data Availability

The original data are available upon request from the corresponding author.
