# Supplementary material for: Leveraging machine learning in precision medicine to unveil organochlorine pesticides as predictive biomarkers for thyroid dysfunction
Source: Sci Rep. 2025 Apr 11;15:12501. doi: 10.1038/s41598-025-94827-z (PMC11992014; doi:10.1038/s41598-025-94827-z)
Supplement: Supplementary file 1 — Supplementary Material 1 [file 41598_2025_94827_MOESM1_ESM.docx]

**Supplementary Material**

**Precision Medicine in Action: Leveraging Machine Learning to Unveil Organochlorine Pesticides as Predictive Biomarkers for Thyroid Dysfunction**

**Samir Shamma^a,b^, Mohamed Ali Hussein^a^, Eslam M. A. El-Nahrery^b^, Ahmed Shahat^b^, Tamer Shoeib^c^, and Anwar Abdelnaser^a*^**

^a^ Institute of Global Health and Human Ecology, School of Sciences and Engineering, The American University in Cairo, New Cairo, 11835, Egypt.

^b^ Department of Chemistry, Faculty of Science, Suez University, Suez, Egypt.

^c^ Department of Chemistry, School of Sciences and Engineering, The American University in Cairo, Cairo, Egypt.

* **Corresponding Author:**

**Anwar Abdelnaser** – Institute of Global Health and Human Ecology, School of Sciences and Engineering, The American University in Cairo, New Cairo, 11835, Egypt; Email: [anwar.abdelnaser@aucegypt.edu](mailto:anwar.abdelnaser@aucegypt.edu), Tel: +20-10-20703410, Fax: +20-2-26152905

| **Table S1.** Our Dataset description | |
| --- | --- |
| Features | Sample count |
| 19 | 230 |

| **Table S2.** Our data attribute types, descriptions, and data types for each attribute. | | |
| --- | --- | --- |
| **Attribute** | **Description** | **Data Type** |
| Age | Age of the patients | Numeric |
| Sex | Sex of the patients | Factor with two levels "f," "m" |
| Thyroid-status | Thyroid status | Factor with two levels: " Normal" and "Abnormal" |
| α-HCH | Concentration of α-HCH | Numeric |
| β-HCH | Concentration of β-HCH | Numeric |
| γ-HCH | Concentration of δ-HCH | Numeric |
| δ-HCH | Concentration of δ -HCH | Numeric |
| Heptachlor | Concentration of Heptachlor | Numeric |
| Heptachlor epoxide | Concentration of Heptachlor-epoxide | Numeric |
| Aldrin | Concentration of Aldrin | Numeric |
| Dieldrin | Concentration of Dieldrin | Numeric |
| Endrin | Concentration of Endrin | Numeric |
| Endrin-Aldehyde | Concentration of Endrin-Aldehyde | Numeric |
| α-endosulfan | Concentration of α-endosulfan | Numeric |
| β-endosulfan | Concentration of β-endosulfan | Numeric |
| *p,p* -DDD | Concentration of *p,p*-DDD | Numeric |
| *p,p*-DDE | Concentration of *p,p*-DDE | Numeric |
| *p,p*-DDT | Concentration of *p,p*-DDT | Numeric |
| Methoxychlor | Concentration of Methoxychlor | Numeric |

| **Table S3.** The performance metrics across different “mtry” values. | | | |
| --- | --- | --- | --- |
| mtry | ROC | Sens | Spec |
| 2 | 0.586116 | 0.746212 | 0.370833 |
| 6 | 0.596812 | 0.696212 | 0.405556 |
| 8 | 0.590751 | 0.671212 | 0.373611 |
| 10 | 0.607281 | 0.670455 | 0.395833 |
| 12 | 0.592293 | 0.679546 | 0.361111 |

| **Table S4.** The hyperparameter tuning performance metrics across different “C” and “sigma” values. | | | | |
| --- | --- | --- | --- | --- |
| C | sigma | ROC | Sens | Spec |
| 0.1 | 0.01 | 0.631524 | 0.887879 | 0.204167 |
| 0.1 | 0.05 | 0.583197 | 0.789394 | 0.316667 |
| 0.1 | 0.1 | 0.600189 | 0.864394 | 0.290278 |
| 1 | 0.01 | 0.630682 | 0.879546 | 0.145833 |
| 1 | 0.05 | 0.644108 | 0.789394 | 0.316667 |
| 1 | 0.1 | 0.627767 | 0.814394 | 0.269444 |
| 10 | 0.01 | 0.657965 | 0.889394 | 0.1625 |
| 10 | 0.05 | 0.588436 | 0.856818 | 0.226389 |
| 10 | 0.1 | 0.60504 | 0.890152 | 0.168056 |

| **Table S5.** The number of target classes for training and testing sets and the ranges for hyper-parameter tuning. | | |
| --- | --- | --- |
| **Class** | **Hyper-Parameters** | **Tuning Range** |
| Logistic Regression | LASSO regularization parameter | Determined using cross-validation within the “cv. glmnet” function |
| RF | “mtry” | “mtry” = [2, 4, 6, 8, 10, 12] |
| SVM | “C” and “sigma “ | “kernel” = ['radial'], “C” = [0.1 to 10.0], and “sigma” = [0.01, 0.05, 0.1] |
| XGBoost | (“nrounds”), (“max_depth”), (“eta”), (“gamma”), (“colsample_bytree”), (“min_child_weight”), and (“subsample”) | “nrounds” = 300, “max_depth” = [3, 6, 9], eta= [0.01, 0.1, 0.3], “gamma” = [0, 1, 5], “colsample_bytree” = [0.5, 0.7, 1], “min_child_weight” = [1, 3, 5], and “subsample” = [0.5, 0.7, 1] |
| GBM | (“n.trees”), (“interaction. depth”), (“shrinkage”), and (“n.minobsinnode”) | “n.trees”= [50, 100, 150], “interaction.depth”=[1, 3, 5], “shrinkage”=[0.01, 0.1, 0.3], and “n.minobsinnode”= [10, 20] |
|  |  |  |

| **Table S6** depicts the median values and interquartile ranges for 16 OCPs. | | | | |
| --- | --- | --- | --- | --- |
| Organochlorine | Min | Max | Mean | Median |
| α-HCH | ND | 4806 | 923 | 324 |
| β-HCH | ND | 3464 | 647 | 482 |
| γ-HCH | ND | 2541 | 435 | 391 |
| δ-HCH | ND | 2053 | 77 | ND |
| Heptachlor | ND | 3302 | 443 | 309 |
| Heptachlor epoxide | ND | 3165 | 450 | 370 |
| Aldrin | ND | 2727 | 456 | 313 |
| Dieldrin | ND | 5287 | 422 | 193 |
| Endrin | ND | 3360 | 408 | 315 |
| Endrin Aldehyde | ND | 2023 | 390 | 282 |
| α-endosulfan | ND | 2641 | 348 | 288 |
| β-endosulfan | ND | 2831 | 326 | 224 |
| *p,p* -DDD | ND | 1137 | 25 | ND |
| *p,p* -DDE | ND | 566 | 14 | ND |
| *p,p* -DDT | ND | 1059 | 184 | 160 |
| Methoxychlor | ND | 3016 | 501 | 366 |


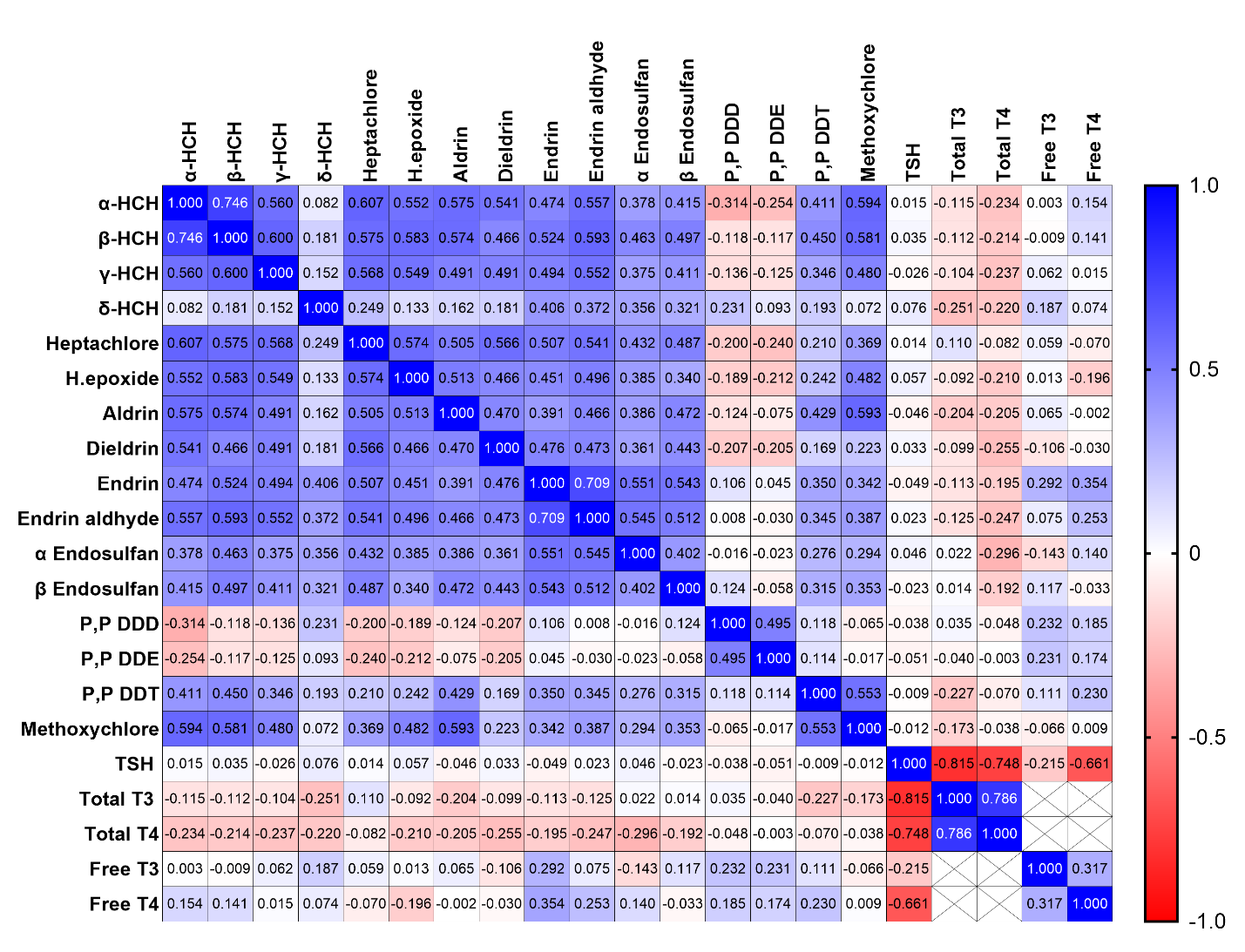


**Fig. S1.** Spearman's correlation relating OCPs level and thyroid hormone level in serum.
